# Epigenetic Regulation of Cellular Senescence

**DOI:** 10.3390/cells11040672

**Published:** 2022-02-15

**Authors:** Jack Crouch, Maria Shvedova, Rex Jeya Rajkumar Samdavid Thanapaul, Vladimir Botchkarev, Daniel Roh

**Affiliations:** 1 Division of Plastic and Reconstructive Surgery, Department of Surgery, Boston University School of Medicine, Boston, MA 02118, USA; jcrouch@bu.edu (J.C.); mariashv@bu.edu (M.S.); rex@bu.edu (R.J.R.S.T.); 2 Department of Dermatology, Boston University School of Medicine, Boston, MA 02118, USA; vladbotc@bu.edu

**Keywords:** senescence, epigenetics, aging, SASP, histone modification, DNA methylation

## Abstract

Senescence is a complex cellular stress response that abolishes proliferative capacity and generates a unique secretory pattern that is implicated in organismal aging and age-related disease. How a cell transitions to a senescent state is multifactorial and often requires transcriptional regulation of multiple genes. Epigenetic alterations to DNA and chromatin are powerful regulators of genome architecture and gene expression, and they play a crucial role in mediating the induction and maintenance of senescence. This review will highlight the changes in chromatin, DNA methylation, and histone alterations that establish and maintain cellular senescence, alongside the specific epigenetic regulation of the senescence-associated secretory phenotype (SASP).

## 1. Introduction

Cellular senescence is characterized as a hallmark of biological and chronological aging and a potential indicator of pathological tissue states [1]. Senescence also occurs in replicating cells at risk for neoplastic transformation and forces exiting of the cell cycle as a preventative measure against uncontrolled proliferation [2]. More recently, cellular senescence has also been shown to mediate normal wound repair and tissue regeneration, demonstrating its importance in maintaining organismal homeostasis during specific stress challenges [3,4]. Indeed, the expression of p16^INK4a^, a cyclin-dependent kinase inhibitor that blocks cell-cycle progression from the G1 to S phase, is rapidly induced upon wounding, and clearance of these p16+ senescent cells from wound sites significantly delays healing [3,5]. Further, senescent cell populations have been identified throughout embryogenic development in mammalian species, including appropriate development of the inner ear [6] and the mesonephros excretory organ, prior to its transition to the metanephros [7]. Recent literature has suggested widespread epigenetic changes in senescent cells are crucial to the induction, progression, and maintenance of senescence. This review will highlight some of the epigenetic mechanisms mediating cellular senescence.

## 2. Cellular Senescence

Cellular senescence was first described in 1961 when Hayflick and Morehead discovered human lung fibroblasts terminally exit the cell cycle after serial passaging. This phenomenon has since been termed the Hayflick limit [8]. This form of cellular senescence, known as replicative senescence, is mediated primarily by telomere shortening [9]. Since the discovery of cellular senescence, many additional mechanisms that trigger the transition to senescence have been described. In addition to replicative senescence, senescence may be induced via aberrant activation of oncogenes such as BRAF^V600E^ or RAS, which mediate cell proliferation and are known drivers of tumor progression and metastasis [10,11]. This oncogene-induced senescence (OIS) drives increased expression of genes for proteins involved in cellular respiration, leading to increased generation of reactive oxygen species [12]. Escalated levels of reactive oxygen species ultimately result in DNA damage and double-stranded DNA breaks (DSB), which trigger the cell to senesce. DSBs trigger senescence by activating a signaling pathway that results in cell cycle arrest. Briefly, DSBs are sensed by ataxia telangiectasia mutated (ATM) protein, which stabilizes p53, facilitating its binding to the *CDKN1A* promoter driving translation of p21, a cyclin-dependent kinase inhibitor that prevents cell-cycle progression [13]. In addition, p16 coordinates with p21 in a p53-independent senescence pathway to induce hypophosphorylation of retinoblastoma protein (Rb) which permanently arrests cells in the G1 phase [14]. Mitochondrial dysfunction, driven by mitochondrial DNA depletion, electron transport chain inhibition, or mitochondrial chaperone depletion also drives cellular senescence [15]. Interestingly, investigators demonstrated this senescence was induced independent of DNA damage, a common senescence trigger. Instead, cellular senescence was mediated by AMP-activated protein kinase (AMPK) detection of decreased carbon source availability, driving activation of p53. Further, these senescent cells had a slightly altered SASP secretome compared to replicatively senescent cells, including loss of IL-6, a common SASP factor. While senescent cell-cycle arrest may appear to be a terminal fate, several studies have driven senescent cells to re-enter the cell cycle through conditional knockdown of proteins critical in the promotion and maintenance of senescence [16,17,18]. These data suggest senescent cell-cycle arrest exists on a continuum, dependent on internal cellular programs and environmental cues.

**Figure 1:**
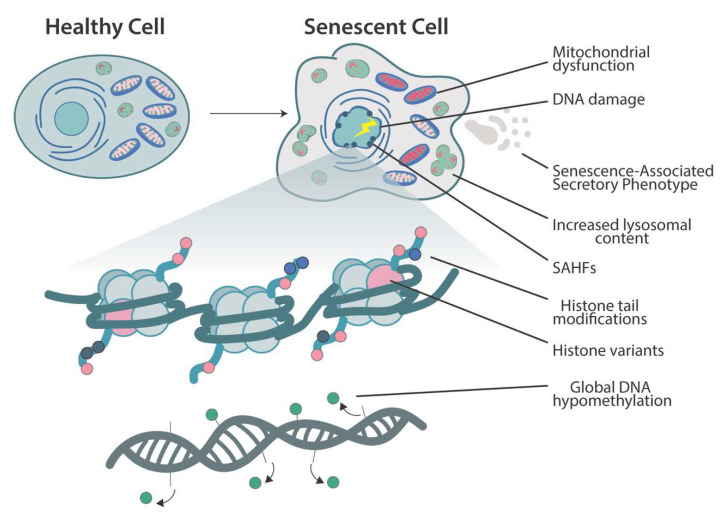
An overview of the morphological, cellular, and epigenomic changes that occur as cell senescence. Morphological alterations include cellular flattening and enlargement. Cellular lysosomal content increases concomitantly with the elevated expression of senescence-associated β-galactosidase, a highly specific marker for senescence [19]. Mitochondrial abnormalities include impaired biogenesis and mitophagy along with increased reactive oxygen species production and decreased membrane potential [20]. Senescent cells also secrete a heterogeneous group of proinflammatory cytokines, chemokines, proteases, and growth factors that have a profound impact on neighboring cells, known as senescence-associated secretory phenotype (SASP). Large-scale chromatin reorganization occurs with the generation of senescence-associated heterochromatin foci (SAHF), which specifically suppress transcription of pro-proliferation genes [23]. Global hypomethylation of DNA is observed in replicative senescence [29]. Histone variant deposition, histone tail acetylation/methylation, and DNA methylation encompass many of the epigenomic alterations that initiate and maintain cellular senescence.

Senescent cells undergo dramatic phenotypic changes (Figure 1). Morphological changes include flattening and enlargement of senescent cell shape and size. The senescence-associated β-galactosidase (SA-β-gal) is a lysosomal enzyme encoded by the gene *GLB1*, which accumulates specifically in senescent cells along with the increase in lysosomal content [19]. Impaired biogenesis and mitophagy, alongside decreased membrane potential and increased reactive oxygen species production, are hallmarks of mitochondria in senescent cells [20]. Senescent cells are highly resistant to apoptosis, which may have clinical implications due to their observed accumulation in varied disease states [21,22]. Another unique phenotype to senescent cells is the formation of senescence-associated heterochromatin foci (SAHF), which are specialized regions of facultative heterochromatin that decrease expression of proliferation-promoting genes [23] (see senescence-associated heterochromatin foci section below). Disruption of SAHF integrity was shown to facilitate cell transformation, demonstrating a clear role in tumor suppression via stable suppression of cell-proliferation-associated genes [24]. Nuclear architecture is also disturbed during aging and senescence. The nuclear lamina, a meshwork of proteins just inside the nuclear envelope, provides the nucleus support and stability to maintain its shape and prevent rupture of the membrane. Lamin B1, a key structural protein of the nuclear lamina, decreases in senescent fibroblasts in vitro [25] and in aged human hippocampal astrocytes [26]. Changes in lamin expression and function are just one component in the complex nuclear architectural changes during senescence and aging. For more detail, please refer to recently published reviews [27,28].

Perhaps the most clinically relevant phenotype of senescent cells is the upregulation of senescence-associated secretory phenotype (SASP) genes, a group of proinflammatory cytokines, chemokines, proteases, and growth factors that can drive tissue dysfunction and carcinogenesis [30]. SASP factors, such as IL-6, IL-8, MCP-1, VEGF, and TGF-β, have been shown to mediate epithelial cell proliferation and have been implicated in the progression and development of various carcinomas [30,31,32]. Increased secretion of IL-8 by senescent fibroblasts stimulated pancreatic cancer invasion and metastasis in in vitro cell culture and in xenograft mouse models. Further, increased IL-8 production is significantly associated with poorer prognosis for patients with pancreatic cancer [33]. Paradoxically, some secretory elements of the SASP, including TGF-β, an immunosuppressive cytokine [34], can induce senescence via paracrine signaling to nearby cells, suggesting the SASP secretome is highly regulated with heterogeneous inputs driving different phenotypes [35,36].

It is well documented that senescent cells accumulate in aged tissues [37] and are associated with tissue/organ pathology, as well as various disease states [38,39,40]. The accumulation of senescent cells in tissues is believed to exhaust proliferation-competent cells alongside renewable stem cells, eventually disrupting the functional and regenerative capacity of the tissue [5,41]. Elevated p16^INK4A^ expression was shown to decrease stem cell regeneration across multiple tissue compartments in mice [42,43].

Another feature of aged and senescent cells is a dysregulated metabolome. Recent evidence has demonstrated that nutrient metabolites play an important role in mediating epigenetic enzymatic activity, regulating aging processes, and acting as potent cell signaling agents [44]. Their role in the epigenetic regulation of senescence and aging is important but beyond the scope of this review. For further information, please see recently published reviews [44,45]. In addition, many of these metabolites are produced via mitochondrial processes, implicating that these organelles likely play a significant role in regulating epigenome modifying proteins. An ongoing area of research will be to explore how mitochondrial dysfunction, epigenetic enzymes, and cellular senescence are integrated. To examine the current literature on this topic, please refer to recent studies [46,47,48,49].

## 3. Chromatin Changes and Histone-Modifying Enzymes in Senescence

Eukaryotic chromosomes are generally organized into two architecturally distinct domains: heterochromatin and euchromatin. Heterochromatin is characterized by its relative inaccessibility to transcription factors due to the compact structure of the chromatin. Conversely, euchromatin contains permissive and relaxed regions of DNA that allow for active transcription [50]. Heterochromatin loss and instability are observed in aging, driving increased transcriptional availability of previously repressed genes in multiple organisms, including humans, *C. elegans*, and *S. cerevisiae* [51,52,53,54]. Premature aging syndromes, including Werner’s syndrome and Hutchinson–Gilford progeria syndrome, are characterized by altered structure and loss of heterochromatin [55,56]. In senescent cells, this heterochromatin loss can be partially attributed to persistent DNA damage that drives decreased histone chaperone protein production and decreased histone biosynthesis [57]. Ivanov et al. demonstrated senescent cells bud off pieces of chromatin from the nucleus, known as cytoplasmic chromatin fragments, into the cytoplasm, where they are phagocytosed and degraded by lysosomes, further contributing to senescent cell heterochromatin loss [58].

The many post-translational modifications of histones play fundamental roles in gene expression and alter the structure of chromatin. Histone acetyltransferases (HATs) catalyze the addition of a negatively charged acetyl group to positively charged lysine residues on histone tails protruding from the nucleosome. This mark destabilizes electrostatic interactions between the DNA wrapped around histone cores, allowing for transcription and increased gene expression. Conversely, removal of the acetyl group by histone deacetylases (HDAC) regenerates the stabilizing electrostatic interactions, decreasing transcription and gene expression. Methylation of histone lysine and arginine residues is another gene-regulating mark. Histone methylation is complex in that residues may be mono-, di-, or trimethylated, and these marks may activate or repress gene transcription [59]. Indeed, di- and trimethylation of H3K4, H3K36, and H3K79 are strongly correlated with active gene transcription, whereas H3K9 and H3K27 methylations repress transcription [60]. There are many other physiologically relevant post-translational histone modifications, including phosphorylation, deimination, ADP-ribosylation, and ubiquitylation that are available for review [59].

### 3.1. Polycomb-Group Proteins and Other Histone-Modifying Enzymes

Polycomb-group (PcG) proteins are highly conserved epigenetic chromatin modifiers that govern stem cell fate [61], are potent regulators of the cell cycle [62], and are upregulated in many cancers [63,64,65,66]. Polycomb repressor complexes (PRC) are a specific subset of PcG proteins expressed in proliferating cells and act to repress expression of senescence-associated genes [67,68,69]. Investigators demonstrated critical recruitment of PRCs by scaffold-attachment factor A (SAFA), a heterogeneous nuclear ribonucleoprotein with diverse functions [70,71,72], to repress transcription of senescence-associated genes within proliferating cells [73]. The long noncoding RNA, P21-associated noncoding RNA DNA damage-activated (PANDA), has a similar role as SAFA, as depletion within proliferating fibroblasts’ stimulated senescence, suggesting important roles in PRC-mediated senescence suppression. Interestingly, in senescent cells, there is a unique interaction between PANDA and nuclear transcription factor Y subunit alpha (NF-YA), a pro-proliferation transcription factor where PANDA acts as a molecular trap, sequestering NF-YA from its normal gene targets, thus decreasing expression. In turn, depletion of PANDA in senescent cells allows for NF-YA to facilitate transcription of proliferation-promoting genes, causing cells to exit senescence and regain proliferative capacity. The regulation of senescence by noncoding RNAs is beyond the scope of this review but has been extensively described in the following reviews [74,75,76].

Deletion of the polycomb repressive complex 2 (PRC2) genes in mice results in embryonic lethality [77], while overexpression of B cell-specific Moloney murine leukemia virus integration site 1 (BMI1), a PRC1 protein, downregulates tumor suppressors p16 and p19^Arf^, driving neoplastic transformation [62]. Recently, investigators demonstrated knockdown of PcG protein, enhancer of zeste homolog 2 (EZH2), an H3K27 methyltransferase, was sufficient to induce senescence and SASP via DNA replication-dependent DNA damage with concomitant p53/p21 induction. They further demonstrated that inducing loss of H3K27me3 marks a transcription repressive trimethylation of the 27th lysine residue of histone H3, via an EZH2 small-molecule inhibitor was sufficient to elicit cellular senescence even with normal EZH2 protein levels. Interestingly, this second mechanism was shown to prompt senescence without DNA damage or p21 expression. Instead, treatment with the EZH2 inhibitor facilitated senescence via activation of the p16 pathway [78]. Jumonji domain-containing protein 3 (JMJD3), an H3K27 demethylase, has been shown to induce cellular senescence via activation of *Ink4a*, driving p16-dependent arrest in the setting of OIS [79], suggesting a role in tumor suppression.

### 3.2. Histone Acetylation

Histone deacetylase 4 (HDAC4), a deacetylase with diverse cellular functions [80], has roles in senescence regulation. HDAC4 expression was shown to be downregulated similarly in both replication-dependent and oncogene-induced senescent cells [81]. This study examined HDAC4’s role in allowing cellular subversion of senescence by knocking out HDAC4 in BJ/*hTERT/Ras/E1A* cells, which are optimized for senescence bypass through oncogenic transformation. Knockout of HDAC4 promoted senescence of these cells through upregulation of senescence-associated genes, as well as increased SA-β-galactosidase activity, a highly specific marker for senescence. Further, eliminating HDAC4 in leiomyosarcomas, cancers known for high expression of HDAC4, led to senescence, arrested proliferation, and enriched SASP factors. In a later study, these investigators demonstrated specific HDAC4/H3K27ac interactions at senescence super-enhancer regions via ChIP-seq [82], solidifying HDAC4’s role as a supervisor of the senescence program. shRNA knockdown of p300, a HAT, significantly delayed replicative senescence in fibroblasts and displayed fewer telomere dysfunction-induced foci, which indicate decreased DNA damage [83]. However, overexpression of p300 was insufficient to drive premature senescence. The study further elucidated p300’s role in regulating senescence by demonstrating that p300 depletion drove a significant decrease in expression of super-enhancer target genes. Finally, ChIP-seq experiments demonstrated direct interactions between p300 and the senescence-associated super-enhancer regions establishing p300 as the main driver of senescence-related acetylation at these regions.

### 3.3. Senescence-Associated Heterochromatin Foci

Senescence drives specialized regions of facultative heterochromatin, which silences proliferation-promoting genes, forming senescence-associated heterochromatin foci (SAHF) [23]. SAHFs have been characterized as a multilayer concentric structure with repressive H3K9me3 marks localizing to a “core” and H3K27me3 marks, forming a “ring” around said core. Interestingly, these repressive marks are not required for the formation of the SAHF, suggesting that deposition of these marks is an independent event from the heterochromatin compaction [84]. SAHF-positive senescent cells have also been found to be completely devoid of linker histone H1, a major protein involved in the stabilization of both DNA wrapped around the nucleosome and chromatin fibers between nucleosomes [85]. Depletion of suppressor of variegation 3–9 homolog 1 (SUV39h1), a histone H3 lysine 9 methyltransferase and a critical enzyme in SAHF formation, promoted tumor progression in a neuroblastoma Ras-induced oncogene transgenic mouse model, suggesting that SAHF are important in preventing neoplastic transformation [86]. After treatment with cell-cycle inhibitors or DNA-damaging agents, ATP-dependent helicase (ATRX), a chromatin-remodeling enzyme, was found to be critical for the development of the SAHF via interactions with H3K9me3 and heterochromatin protein 1 gamma (HP1γ), a nonhistone chromatin-binding protein that drives gene suppression. Further, knockdown of ATRX prevented formation of SAHF in vitro [87]. Investigators demonstrated an Rb-deficient breast cancer cell line fails to form SAHF following treatment with the DNA-damaging agent. Further, knockdown of Rb in a cancer cell line inhibited SAHF formation in response to doxorubicin, suggesting Rb may play a role in tumor cell SAHF formation after treatment with DNA-damaging agents [88]. These investigators went on to demonstrate that JMJD3 is transported to the cytoplasm and demethylates Rb, facilitating assembly of the SAHF [89]. Importantly, the data discussed above were performed in vitro utilizing human fibroblast cell lines. Recent literature has demonstrated that SAHF formation is dependent on cell type and the senescence induction method. Lentiviral oncogene induction drove SAHF formation in two fibroblast cell lines and keratinocytes. However, exposure to cytotoxic chemotherapeutics, bacterial toxins known to induce senescence, and serial passaging only induced SAHF formation in some fibroblast cell lines and did not induce SAHF in keratinocytes [90]. Further, the authors demonstrated that SAHFs were not detectable in β-galactosidase-positive cells of healthy human bladder and colon tissues alongside urinary bladder and colorectal tumors. The data above suggest that SAHF formation is not a universal mechanism of senescence and is tissue type and senescence induction method dependent.

### 3.4. Histone Variants

Histone variants of the canonical histone proteins H2A, H2B, H3, and H4 have crucial roles in transcriptional regulation, chromosome segregation, DNA repair, and generation and maintenance of epigenetic states [91]. While most canonical histones are deposited during the S phase of cellular replication, variants can be deposited in a replication-dependent or -independent manner [91]. Replication-induced senescent cells are characterized by a variety of canonical histone replacements by histone variants. H3 is cleaved by lysosomal protease cathepsin L1 (CTSL1), after its localization to the nucleus via Ras activation, to H3.3, and CTSL1 inhibition prevents formation of the SAHF. Further, ectopic expression of H3.3 induced senescence in fibroblasts, demonstrating H3.3’s role in senescence-specific heterochromatin alterations [92]. H3.3 chromatin deposition is mediated by histone chaperone HIRA. Coordination of HIRA and H3.3 is critical for senescence induction, as investigators demonstrated large-scale neoplastic transformation in an oncogenic *Braf* mouse model via inactivation of HIRA [93]. SAHFs are enriched with another histone variant, macroH2A. Indeed, HIRA/antisilencing function 1A Histone chaperone (ASF1A)-mediated macroH2A deposition is necessary for the stable formation of the SAHF and was determined to be the rate-limiting step in SAHF formation [94]. Another variant, H2A.J, was found to accumulate in senescent human fibroblasts and aged mice in the setting of persistent DNA damage. Knockdown of H2A.J decreased expression of common SASP-associated genes, and overexpression of H2A.J in nonsenescent proliferating cells increased expression of SASP-associated genes, highlighting the importance of this histone variant in facilitating the senescence phenotype [95].

Unlike previously mentioned variants that facilitate and maintain senescence, H2A.Z is a potent suppressor of senescence induction in proliferating cells by localizing to p53 binding sites at the p21 promoter. Upon treatment with a DNA-damaging agent, ChIP assay confirmed eviction of H2A.Z from the p21 promoter, allowing p53 binding and inducing cellular senescence. Further, depletion of H2A.Z from primary fibroblasts was sufficient to induce senescence [96].

γ-H2AX, which denotes phosphorylation of the Ser-139 residue of the histone variant H2AX, is a histone variant that is present at double-stranded DNA breaks (DSB) where it colocalizes to the DSB, inducing a rapid epigenetic response to drive resolution of the break [9]. Further, γ-H2AX foci are widely observed in the nuclei of senescent cells and cells that have undergone significant telomere shortening [97].

Altogether, variant histones are highly abundant in senescent cells relative to their proliferating cell counterparts, stabilizing and maintaining SAHFs, as well as facilitating SASP factor gene expression, allowing for sustained cellular senescence.

## 4. DNA Methylation

DNA methylation is a powerful epigenetic regulatory modification that promotes transcriptional silencing. In replicative senescence, an overall decrease in DNA methylation was observed in senescent cells compared to proliferating cells. This hypomethylation was attributed to a failure of DNA methyltransferase 1 (DNMT1) to maintain methylation over many repeated replication cycles, which drives decreased expression of DNMT1 [29] and delocalization of the enzyme out of the nucleus [98]. Methylation patterns vary across types of senescence, as demonstrated by Sakaki et al. Their investigation revealed altered DNA methylation patterns in replicative senescent cells compared to their proliferating cell counterparts. Hypomethylation was specifically enriched at CpG sites related to immune response genes. Interestingly, little change was observed in methylation patterns for premature induction of senescence via Ras-induced oncogenic stress. Cellular senescence induction via oncogenic stress in cells that had undergone several rounds of replication recapitulated the methylation patterns observed in replicative senescent cells. Further, hypomethylation was enriched by nonCpG island promoters of immune response-related genes, suggesting a role in the upregulation of certain SASP genes [99].

DNA methylation and subsequent oxidation of 5-methylcytosine (5-mC) into 5-hydroxymethylcytosine (5hmC) are key epigenetic events regulating development and stem cell differentiation in mammals [100,101,102]. The process of 5-mC conversion toward an unmethylated state also includes formation of 5-formylcytosine and 5-carboxycytosine [103]. Oxidation of 5-mC is catalyzed by the ten-eleven translocation (Tet) family enzymes (Tet1/2/3) and serve as an important step in DNA demethylation [104]. In addition to 5-mC oxidation at gene regulatory regions and CpG islands, Tet proteins are also capable of positively regulating gene transcription by activating enhancers via DNA demethylation [102,105].

Increasing evidence suggests that Tet enzymes contribute to aging of reproductive and hematopoietic systems. *Tet1* deficiency reduces fertility and leads to accelerated reproductive failure (premature ovarian insufficiency) syndrome with age [106]. Tet1-deficient mice undergo a progressive reduction of spermatogonia stem cells and spermatogenesis resulting in accelerated infertility with age [107]. Somatic *TET2* mutations contribute to myeloid expansion and innate immune dysregulation with age accompanied by an increased risk of transformation to hematological malignancies, including myeloproliferative and myelodysplastic neoplasms and acute myeloid leukemia [108].

An “epigenetic clock” has been proposed via next-generation sequencing technology that describes highly reproducible patterns of methylation that can accurately predict human chronological age [109]. Further, this DNA methylation clock has been established across multiple murine strains, including many tissue types [110]. Cell passage numbers and population doublings of in vitro cell cultures may also be predicted via analysis of methylation patterns to specific CpG sites [111]. Age-associated and senescence-associated changes in DNA methylation share considerable overlap, but do harbor distinct differences at specific CpG sites, most commonly found at *Hox* genes and genes mediating cellular differentiation [112]. The similarities observed suggest both aging and senescence are regulated by similar mechanisms. Previous reports also demonstrated striking similarities between the global methylation profile of senescent cells and those of cancer cells [98,113], suggesting premalignant senescent cells may facilitate malignant transformation if senescence is bypassed. However, a more recent analysis of the senescent cell methylation demonstrated key local differences in methylation events between replicative senescence and transformation [114]. Indeed, in senescent cells, programmed methylation of CpGs was found in promoter regions of genes regulating metabolism and cellular biosynthesis. Conversely, stochastic methylation of genes regulating cell differentiation and development was found in transformed cells that may drive self-renewal and block differentiation.

Recent studies further interrogated the epigenetic clock to recapitulate a juvenile epigenetic profile in aged cells. Investigators transfected aged primary human fibroblasts and endothelial cells with an mRNA cocktail expressing OSKMLN, the canonical induced pluripotent stem cell (iPSC) reprogramming factors. This specific methodology allows for epigenetic reprogramming without altering the original cell identity. After treatment, aged primary cells exhibited a rapid reversion of their epigenetic clock methylation pattern, appearing similar to their young cell counterparts. Immunofluorescence data further demonstrated a youthful epigenetic profile with increased nuclear staining of H3K9me3, HP1γ, and lamin-associated polypeptide 2α (LAP2α), a nuclear lamin support protein. Further evidence of improved cellular function included increased SIRT1 expression, decreased mitochondrial reactive oxygen species, and improved active clearance of cellular waste and macromolecules. [115]. This model has also been utilized in replicatively senescent human donor fibroblasts and human cell line fibroblasts [116]. Lapasset et al. showed in both groups of cells decreased markers of senescence, including disappearance of SAHFs, downregulated expression of p16^INK4A^ and p21^CIP1^, increased telomere length, and restoration of proliferation capacity. In vivo reprogramming was also used in the LAKI progeroid mouse model, which harbors a mutation in the lamin A gene. Short-term cyclic expression of OSKM restored heterochromatin maintenance marks H3K9me3 and H4K20me3 to healthy levels. Liver cells isolated from these mice harbored significantly fewer SA-β-Gal positive cells compared to control mice. Furthermore, fibroblasts harvested from these progeroid mice that underwent reprogramming demonstrated increased mitochondrial function, reduced cellular senescence evidenced by *Il-6* and β-galactosidase expression, and decreased DNA damage [117]. This transfection method was used to restore youthful DNA methylation patterns and transcriptomes in aged mouse CNS tissue leading to increased axon regeneration after injury and restored vision loss in an aged mouse glaucoma model [118].

Altogether, alterations in DNA methylation and DNA methylation associated enzymes during aging and senescence significantly contribute to cellular dysfunction, facilitate a chronic inflammatory state via SASP activation, and may mediate certain age-related pathologies.

## 5. Epigenetic Regulation of SASP

**Figure 2:**
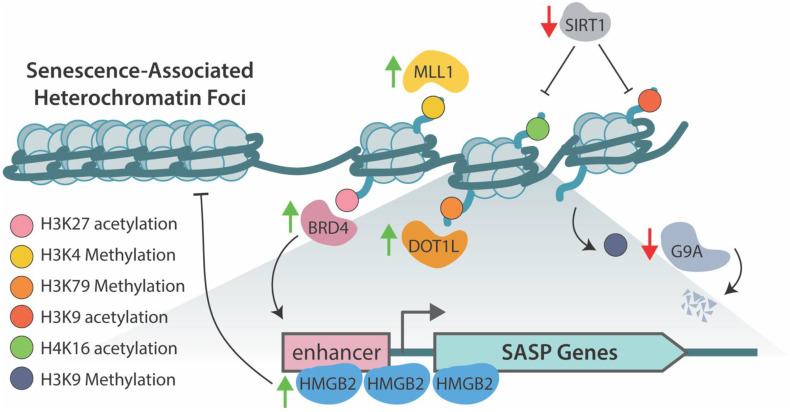
An overview of the histone acetylation/methylation marks and proteins that regulate genes involved in the senescence-associated secretory phenotype (SASP). High-mobility-group protein B2 (HMGB2) binds loci of key SASP genes preventing their incorporation into the SAHF during senescence allowing for their transcription [120]. Mixed-lineage leukemia 1 (MLL1) methylates H3 at Lys-4 to promote transcription of SASP genes [122]. Disruptor of telomeric silencing 1-like (DOT1L) mediates methylation of H3 at Lys-79 driving transcription of potent inflammatory cytokine and SASP factor *IL1A* [123]. Bromodomain-containing protein 4 (BRD4) mediated acetylation of H3 at Lys-27 is necessary to drive transcription of SASP factors during senescence [121]. G9a and G9a-like proteins, which deposit repressive methylation on H3 at Lys-9, prevent transcription of SASP genes in proliferating cells [124]. In response to DNA damage, these proteins are degraded, allowing for active transcription of IL-6 and IL-8. NAD-dependent deacetylase sirtuin-1 (SIRT-1) negatively regulates SASP expression through deacetylation of H4 Lys-16 and H3 Lys-9 in normal proliferating cells. Senescence induction decreases expression of SIRT-1, allowing for acetylation and expression of key SASP genes [126].

High-mobility-group protein B2 (HMGB2), a multifunction protein involved in chromatin remodeling, transcription regulation, and DNA repair [119], binds loci of SASP genes, preventing their incorporation into the SAHF during senescence, thus facilitating active transcription (Figure 2). Inhibition of HMGB2 decreases SASP in senescent cells without preventing formation of the SAHF. Incorporation of the *IL8* gene locus into the SAHF was demonstrated to repress this SASP factor in HMGB2 knockdown senescent cells. Targeting this pathway may allow beneficial repression of neoplastic transformation and proliferation genes via cellular senescence while inhibiting a chronic proinflammatory and protumorigenic environment of SASP factors [120].

Chromatin remodeling events during cellular senescence drive the formation of super-enhancers at SASP gene loci to facilitate transcription. Increasing H3K27 acetylation at these super-enhancer sites and recruitment of Bromodomain-containing protein 4 (BRD4) is necessary for upregulated expression of key SASP genes and inhibition of BRD4 suppressed SASP expression. Further, pharmacological inhibition of BRD4 allowed for immune escape and decreased clearance of premalignant senescent hepatocytes in a liver cancer mouse model [121].

The H3K4 methyltransferase, mixed-lineage leukemia 1 (MLL1), is required for SASP activation, as silencing of MLL1 with shRNA dramatically decreased expression of key SASP genes, including IL-1A, IL-8, IL-6, MMP1, and MMP3m in OIS-transformed fibroblasts and OIS primary human melanocytes. Interestingly, inhibition of MLL1 blocked the expression of SASP-like proteins in response to DNA damage that did not induce senescence, suggesting an anti-inflammatory role independent of senescence [122]. Disruptor of telomeric silencing 1-like (DOT1L), a histone-specific methyltransferase, was shown to be necessary for methylation of H3K79 at the *IL1A* gene locus, driving increased transcription. Like MLL1, this phenomenon was independent of senescence-induced cell-cycle arrest, making MLL1 and DOT1L potential therapeutic targets to limit the negative consequences of SASP factors of the tissue environment without losing the antineoplastic transformation effect of senescence [123].

The DDR during senescence promotes proteasomal degradation of G9a and G9a-like protein, both H3K9 histone methyltransferases. In senescence-induced cells, Cdc14B- and p21-dependent activation of APC/C ubiquitin ligase drive decreased H3K9 methylation, allowing for increased expression of IL-6 and IL-8 [124]. JMJD3 was found to be upregulated in several transformed glioma cell lines compared to healthy astrocytes. Further, ectopic overexpression of JMJD3 within a glioma cell line promoted SASP-associated gene expression and induced senescence suggesting JMJD3’s demethylase activity mediates SASP activation [125].

NAD-dependent deacetylase sirtuin-1 (SIRT-1) negatively regulates SASP genes. During cellular senescence, decreased expression of SIRT-1 allows for increased acetylation of H3K9 and H4K16 driving transcription of these cytokines, a phenomenon recapitulated in nonsenescent SIRT-1 depleted cells [126]. Interestingly, while DNA damage and senescence are generally inextricably tied, treatment of proliferating cells with sodium butyrate, an HDAC inhibitor, induced senescence and promoted expression of SASP genes in the absence of DNA damage [127,128]. This finding suggests chromatin remodeling due to the DDR pathway may be the significant driver of SASP expression, as opposed to physical breaks within DNA.

## 6. Therapeutics Targeting Epigenetic Mechanisms

As more data implicate epigenetic regulatory mechanisms of gene expression playing an integral role in the manifestation and maintenance of age-related diseases, investigation has turned toward therapies that combat these pathologies alongside promoting health and lifespan. Resveratrol is a sirtuin-1 activator that has improved health and lifespan in many species, including mice [129,130,131], but its clinical efficacy as a therapeutic in humans is less well understood. HDAC inhibitors are a group of therapeutics with diverse structure, specificity, and biological activity that prevent deacetylation of histone tails. Their improvement of healthspan and amelioration of disease states has been well documented in many species, including rodents. HDAC inhibitor treatment improved neuronal health, cognitive function, and memory in neurodegenerative rodent models, including Alzheimer’s disease [132], amyotrophic lateral sclerosis [133], and Parkinson’s disease [134]. HDAC inhibitors also prevented obesity, preserved insulin sensitivity, and improved mitochondrial function in mice fed a high-fat diet compared to their control littermates [135,136]. Currently, five HDAC inhibitors have been approved by the US Food and Drug Administration (FDA), vorinostat, romidepsin, tucidinostat, Panobinostat, and belinostat. Romidepsin and vorinostat are current therapies in the treatment of cutaneous T-cell lymphoma [137,138]. Romidepsin and belinostat are used for treating peripheral T-cell lymphoma [139,140]. DNMT inhibitors are another class of epigenetic regulation targeting drugs that have found efficacy in treating cancer. Azacitidine and decitabine are antimetabolite DNMT inhibitors with efficacy in acute myeloid leukemia, chronic myelomonocytic leukemia, and myelodysplastic syndrome [141]. Tazemetostat is an EZH2 inhibitor that is used in the treatment of non-Hodgkin’s lymphoma [142]. Importantly, these epi-drugs are not without their limitations. As seen above, their use has been primarily restricted to cancers of hematologic origin. Indeed, their efficacy as monotherapies against solid tumor cancers is poor. However, there is growing preclinical evidence that combination therapies of HDAC inhibitors or other epi-drugs paired with other anticancer therapeutics may be more effective in treating solid tumor cancers. For example, tamoxifen paired with an HDAC inhibitor was effective in the treatment of hormone-sensitive breast cancer and helped prevent or reverse cancer resistance [143,144]. Another limitation of epi-drugs is their distinct lack of specificity leading to significant off-target effects and cytotoxicity. Continued study of the histone code is imperative to generate more specific therapies that can maximize effectiveness against cancer cells and minimize toxicity to healthy cells. For more detail on epi-drugs, please see current reviews [145,146,147].

## 7. Conclusions and Future Perspectives/Directions

Drugs targeting epigenetic pathways remain an exciting field of therapeutics; however, they are currently targeted towards hematologic cancers. Continuation of research examining the histone code, epigenetic protein/nucleotide interactions, and combination therapies will improve the efficacy of these drugs, limit unintended effects, and perhaps expand their usefulness therapeutic to additional domains such as senescence and aging. As discussed, epigenetic changes in senescence are diverse and dynamic, and there is no universal phenotype of senescent cells. As epigenetic regulation of senescence is dependent on tissue and cell type, as well as a senescence induction method, there is a need for expansion of the tissue types and cells studied during senescence induction would allow better insight into how different cell types may reorganize chromatin due to various senescence-promoting stimuli.

Large-scale chromatin remodeling, histone alterations, and DNA methylation activate or repress cellular senescence in response to a variety of stimuli, including DNA damage, oncogenic stress, mitochondrial dysfunction, and metabolic stress. Further, some epigenetic phenotypes and pathways may be highly specific to various senescence-inducing sources, with others having dramatic overlap. Young, healthy cells maintain an epigenome that promotes precise regulation of gene expression, thus avoiding replicative and oxidative stress. Conversely, aged cells accumulate stochastic insults over time, driving alterations in gene expression, decreased fitness and functionality, ultimately leading to cell-cycle arrest and senescent states. The reversible capacity of these tightly regulated epigenetic pathways and mechanisms serves as a potential source for therapy in myriad disease states caused by senescence.
